# Efficacy and Safety of Letibotulinum Toxin A for the Treatment of Melasma in Two Different Dilutions: A Randomized Double-Blind Split-Face Study

**DOI:** 10.3390/toxins17070349

**Published:** 2025-07-11

**Authors:** Juthapa Pongklaokam, Woraphong Manuskiatti, Rungsima Wanitphakdeedecha, Pitchaya Maneeprasopchoke, Panwadee Thongjaroensirikul, Yanin Nokdhes, Rona Maria R. Abad-Constantino, Woramate Bhorntarakcharoen, Sariya Sittiwanaruk, Thanya Techapichetvanich

**Affiliations:** 1 Department of Dermatology, Faculty of Medicine Siriraj Hospital, Mahidol University, 2 Wanglang Road, Bangkok Noi, Bangkok 10700, Thailandrungsima.wan@mahidol.ac.th (R.W.); yanin.nokdhes@gmail.com (Y.N.);; 2 Department of Dermatology, Research Institute for Tropical Medicine, 9002 Research, Mutinlupa 1781, Philippines

**Keywords:** melasma, Letibotulinum toxin A, hyperpigmentation

## Abstract

Background: Melasma is an acquired hyperpigmentation disorder with multifactorial etiologies and limited response to conventional therapies. Recent evidence suggests that Botulinum Toxin A (BoNT-A) may modulate ultraviolet (UV)-induced pigmentation and offer therapeutic benefits. Objective: We sought to evaluate the efficacy and safety of two intradermal dilutions of Letibotulinum toxin A (LetiBoNT-A) in Thai patients with melasma. Methods: In this randomized, double-blind, split-face study, 30 participants aged 32–62 years received a single intradermal injection of LetiBoNT-A, with 20 units administered per cheek. A 1:5 dilution was injected on one side of the face, and a 1:10 dilution was injected on the contralateral side. Outcomes were evaluated over a 6-month period using the Hemi-modified Melasma Area and Severity Index (Hemi-mMASI), VISIA^®^ brown spot analysis, and quantitative assessments of skin texture. Results: Both dilutions significantly improved Hemi-mMASI scores (1:5, *p* = 0.043; 1:10, *p* = 0.002) and brown spots (1:5, *p* = 0.002; 1:10, *p* < 0.001). The 1:10 dilution showed earlier and more sustained improvements. Subgroup analysis revealed greater reductions in Hemi-mMASI scores among patients with telangiectatic melasma, particularly with the 1:10 dilution, though they were not statistically significant. Additionally, the 1:10 dilution significantly reduced pore volume, pore area, and sebum levels. One case of transient facial asymmetry was reported with the 1:5 dilution. Conclusions: LetiBoNT-A is a safe and effective adjunct in melasma treatment. The 1:10 dilution offered superior clinical outcomes.

## 1. Introduction

Melasma is an acquired hyperpigmentation disorder presenting as brown to gray/brown facial patches, commonly on sun-exposed areas. It is classified as epidermal, dermal, mixed, or indeterminate [1].

The condition is driven by hyperactive melanocytes and influenced by factors such as UV exposure, hormonal changes, genetics, and medications. Recent studies also implicate mast cell activity, solar elastosis, basement membrane disruption, and vascular changes [2]. UV radiation plays a central role by increasing tyrosinase activity and upregulating pigment-inducing mediators such as VEGF [3,4,5].

Melasma treatment involves sun protection and pigment-suppressing therapies. Topical agents, including hydroquinone, triple-combination creams, azelaic acid, and chemical peels, target melanin synthesis and inflammation [6]. Tranexamic acid, given orally, topically, or intradermally, also acts on vascular and pigment pathways [2]. Laser- and light-based therapies (e.g., Q-switched, picosecond, and fractional lasers) are options for refractory cases but carry risks such as PIH and ochronosis. Oral tranexamic acid may also increase thrombotic risk [1,2,6].

Botulinum Toxin and Skin Pigmentation

Botulinum toxin A (BoNT-A), derived from *Clostridium botulinum*, inhibits acetylcholine release at neuromuscular junctions, causing temporary muscle paralysis [7,8,9]. Of the known serotypes (A–G), only BoNT-A and BoNT-B are used clinically, with BoNT-A being the most widely applied due to its efficacy and duration [10,11,12].

Interest in BoNT-A’s pigment-modulating effects stemmed from reports of localized hypopigmentation following periocular injections in patients with facial spasms [13]. Subsequent in vitro studies demonstrated that BoNT-A pretreatment reduced melanin content, tyrosinase activity, and dendrite formation in UVB-irradiated melanocytes and keratinocytes [14]. Animal studies confirmed reduced pigmentation, tyrosinase activity, and levels of inflammatory mediators such as bFGF, IL-1α, and PGE2 [14].

Clinical studies have reported decreased melanin levels in BoNT-A-treated skin, with improved brightness and reduced pigmentation in both pre- and post-UVB exposure models [15,16,17]. However, one study found no significant effect, suggesting that timing and dosage may influence outcomes [18].

Beyond pigment modulation, BoNT-A improves skin texture and reduces lentigines, pore size, and telangiectasia [19,20]. Its antiangiogenic effects, mediated by VEGF downregulation [21], further support its application in vascular conditions such as rosacea [22,23,24,25,26,27,28,29,30,31,32]. Collectively, these findings suggest BoNT-A as a potential treatment for melasma, addressing both pigmentary and vascular components.

Melasma Clinical Research

While in vitro studies support the antimelanogenic and vascular-modulating effects of botulinum toxin A (BoNT-A), clinical evidence in relation to melasma remains limited. A split-face study in Thailand involving twelve patients with mixed-type melasma demonstrated that intradermal Abobotulinum toxin A (AboBoNT-A) injections in one cheek (0.1 mL per point, spaced 1 cm apart; 2.5 units/cm^2^) led to significant reductions in H-mMASI scores and melanin index on the treated side across all follow-up visits (2, 4, 8, and 12 weeks), while the untreated side showed no change [33].

A more recent randomized, double-blind, split-face study reinforced these findings. Twelve patients received AboBoNT-A at 66.7 units/mL via 2 mm intradermal wheals spaced 2 mm apart. At 3 months, significant improvements were observed in both the Melasma Area and Severity Index (MASI) and Physician Global Assessment (PGA) scores.

Complementary in vitro studies using UVA-irradiated B16F10 melanoma cells confirmed that BoNT-A reduced melanin content and tyrosinase activity in a dose-dependent manner without cytotoxic effects [34].

These clinical and laboratory findings support BoNT-A as a promising treatment for melasma, acting through inhibition of melanogenesis and modulation of inflammatory and vascular pathways.

Significance of this Study

Botulinum toxin shows promise in treating melasma by targeting both pigmentation and vascular components. However, melasma remains difficult to manage due to its multifactorial pathogenesis, recurrence, and variable treatment outcomes. Existing studies on BoNT-A are limited by small sample sizes and lack standardized dilution protocols, and Letibotulinum toxin A (LetiBoNT-A) has not yet been evaluated in this context.

In this study, we aimed to compare the efficacy and safety of two intradermal LetiBoNT-A dilutions (1:5 and 1:10) using a randomized, double-blind, split-face design. Secondary outcomes included changes in pore size, sebum production, and facial erythema. Subgroup analysis was conducted to assess responses between telangiectatic and non-telangiectatic melasma types. The findings may help establish standardized protocols for BoNT-A use in melasma treatment.

## 2. Results

A total of 31 participants were enrolled, but 1 withdrew due to scheduling conflicts. Thus, 30 completed this study and were included in the final analysis.

### 2.1. Demographic Data

The mean age was 47 ± 6.6 years (range: 32–62), with most participants being female (96.67%) and having Fitzpatrick skin types IV (70%) or V (16.67%). Melasma was categorized as non-telangiectatic (53.33%) and telangiectatic (46.67%). Baseline Hemi-mMASI scores were higher in the telangiectatic group across both dilutions. Pain scores were similar between dilutions (1:5 = 5.13 ± 2.29; 1:10 = 5.23 ± 2.21) (Table 1).

### 2.2. Outcomes

Primary outcomes:

1. Hemi-modified Melasma Area and Severity Index (Hemi-mMASI);
2. Brown spot count via VISIA^®^;
3. Adverse events.

Secondary outcomes:

1. Red area via VISIA^®^;
2. Sebum level (Sebumeter^®^);
3. Mean pore area and volume (Antera 3D^®^);
4. Subjective evaluation by dermatologists;
  (Investigator Global Aesthetic Improvement Scale (IGAS));
5. Subjective evaluation by participants (Patient Global Assessment (PGA)).

#### 2.2.1. Mean Hemi-MASI Modified Melasma Area and Severity Index (Hemi-mMASI) in the Melasma Area

**Figure 1:**
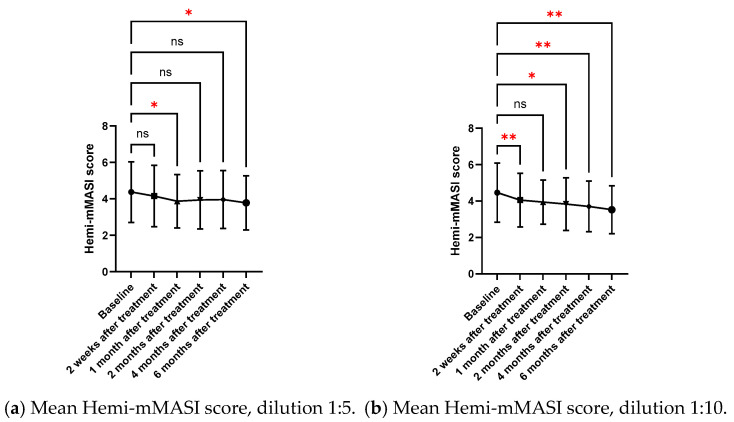
(**a**) Mean Hemi-mMASI score dilution 1:5; (**b**) mean Hemi-mMASI score dilution 1:10. *p* < 0.05 (*), *p* < 0.01 (**); ns = not significant. Statistical comparison versus baseline.

The subjective assessment of Hemi-mMASI scores by two blinded dermatologists revealed significant improvements in melasma severity for both 1:5 and 1:10 dilutions over six months. Statistically significant reductions were noted at specific follow-up points: 2 weeks for the 1:10 dilution (*p* = 0.006), and 6 months for both dilutions (1:5 dilution: *p* = 0.043; 1:10 dilution: *p* = 0.002). The 1:10 dilution showed a more consistent statistical significance reduction in Hemi-mMASI score from baseline compared to the 1:5 dilution (Table 2, Figure 1).

At the 6-month follow-up, the 1:5 dilution resulted in a 14% improvement in Hemi-mMASI scores (from 4.38 ± 1.66 at baseline to 3.78 ± 1.49 at 6 months, *p* = 0.043), while the 1:10 dilution led to a 21% improvement in Hemi-mMASI scores (from 4.47 ± 1.63 at baseline to 3.53 ± 1.32 at 6 months, *p* = 0.002) (Table 2).

However, when comparing the two concentrations directly, no significant differences were found, indicating that both dilutions are similarly effective (Table 2). The 1:10 dilution demonstrated faster and more consistent improvements, with greater reductions in pigmentation at each time point as soon as 2 weeks after injections (*p* = 0.006) and sustainable for 4 and 6 months (*p* = 0.002). Therefore, while both dilutions were effective, the 1:10 dilution provided faster and generally more significant improvements in melasma treatment (Figure 1).

**Figure 2:**
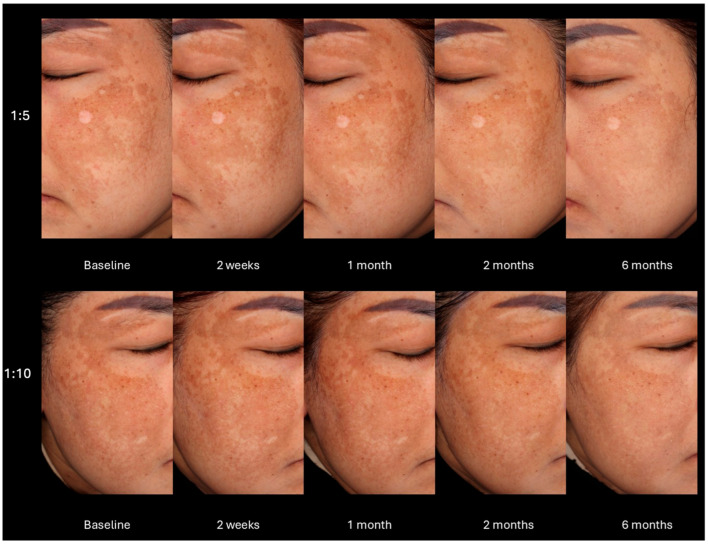
Clinical outcomes after treatment at different dilutions.

Figure 2 presents an example of clinical pictures assessed by the blinded dermatologists, demonstrating improvements aligned with the Hemi-mMASI scores. The 1:10 dilution exhibited notable reduction in pigmentation as early as the 2-week follow-up and sustained continuous improvement by 6 months, revealing even more substantial alterations relative to the baseline. Conversely, the 1:5 dilution exhibited less pronounced enhancements, yielding somewhat less impressive outcomes, but nonetheless indicated significant improvement at 6 months relative to the baseline.

#### 2.2.2. Brown Spots and Red Areas Assessed with VISIA^®^ in the Melasma Area

**Figure 3:**
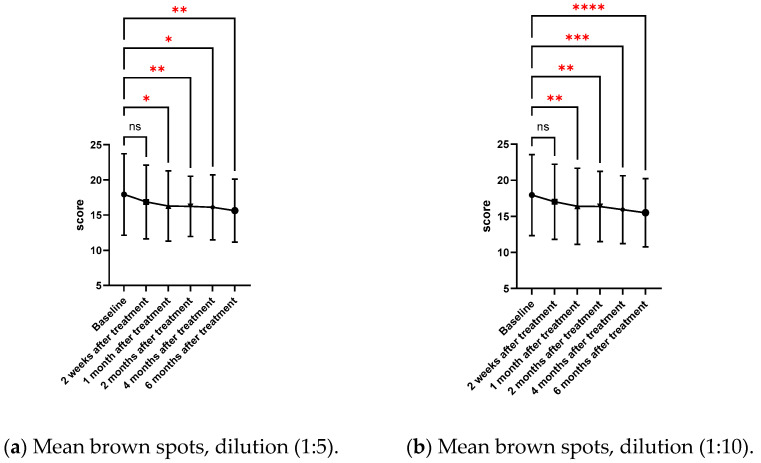

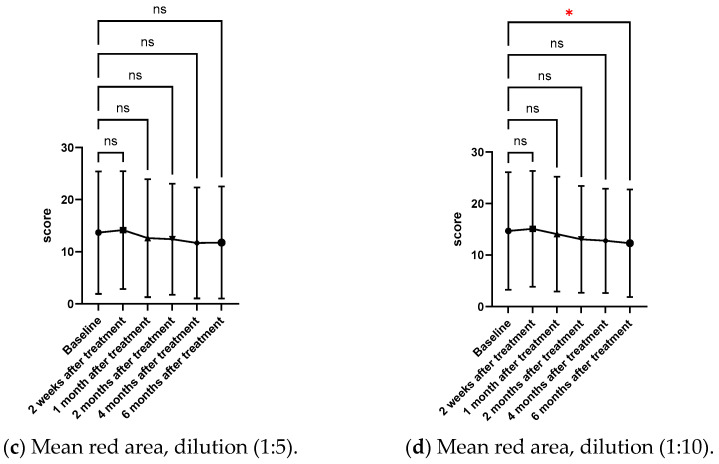
The mean brown spot scores for the 1:5 (**a**) and 1:10 (**b**) dilutions, as well as the mean red area scores for the 1:5 (**c**) and 1:10 (**d**) dilutions, evaluated throughout the treatment period. *p* < 0.05 (*), *p* < 0.01 (**), *p* < 0.001 (***), *p* < 0.0001 (****); ns = not significant. Statistical comparisons were performed versus baseline.

The objective assessment of brown spots and red areas with VISIA^®^ is shown in Table 3 and Figure 3. Over a six-month period, VISIA^®^ complexion analysis demonstrated substantial decreases in brown spots for both dilutions (1:5 and 1:10). Improvements were found to be statistically significant as early as from one month (*p* = 0.017 for 1:5; *p* = 0.007 for 1:10) to six months (*p* = 0.002 for 1:5; *p* < 0.001 for 1:10). For the red area, only the 1:10 dilution shows a significant reduction at 6 months (*p* = 0.032), while this was not seen in the 1:5 dilution.

#### 2.2.3. Subgroup Analysis

A subgroup analysis was performed to evaluate treatment responses between two melasma subtypes: non-telangiectatic melasma (pigment-only type) and telangiectatic melasma (characterized by the presence of visible vascular components). Both groups demonstrated reductions in Hemi-mMASI scores across all follow-up time points for both the 1:5 and 1:10 dilutions. However, no statistically significant differences were observed between the two subtypes at any time point or across any of the measured parameters, including mean change in Hemi-mMASI scores and brown spot counts.

**Figure 4:**
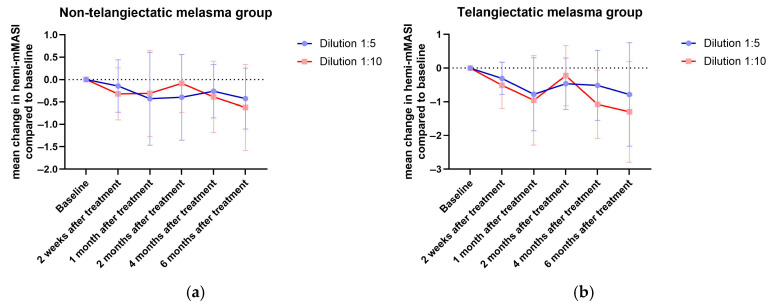
Mean change in Hemi-modified Melasma Area and Severity Index comparing between two dilutions in the (**a**) non-telangiectatic and (**b**) telangiectatic melasma groups.

**Figure 5:**
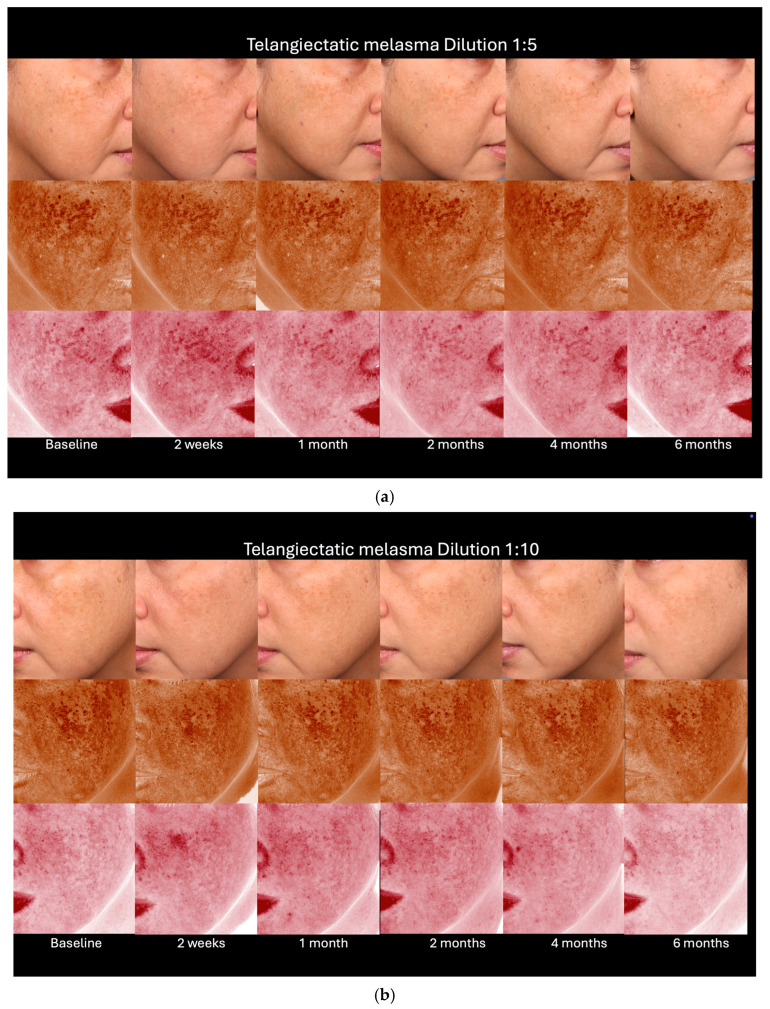
Clinical outcomes of telangiectatic-type melasma assessed with VISIA^®^ imaging (Canfield Scientific, Inc., Parsippany, NJ, USA) following treatment with dilutions 1:5 (**a**) and 1:10 (**b**).

At 6 months (Figure 4 and Figure 5), the telangiectatic melasma group generally showed greater reductions in Hemi-mMASI scores from baseline, particularly with the 1:10 dilution, although these changes did not reach statistical significance. This trend was less apparent with the 1:5 dilution. Despite the absence of statistical differences, the numerical improvement in the telangiectatic group may suggest a slightly enhanced clinical response, warranting further investigation in larger studies.

These findings suggest that the efficacy of LetiBoNT-A was comparable between melasma subtypes, with both pigment-dominant and vascular-associated forms responding similarly to treatment.

#### 2.2.4. Sebum Level from Measurement with Sebumeter^®^ in the Blemish Area

The Sebumeter^®^ assessment revealed differences in sebum levels between the 1:5 and 1:10 dilutions. For the 1:10 dilution, a statistically significant reduction in sebum levels was observed at the 1-month follow-up (*p* = 0.037) compared to the baseline over time (Table S1).

#### 2.2.5. Mean Pore Area and Mean Pore Volume Based on Antera 3D^®^

This study evaluated the mean pore volume and area using Antera^®^ 3D (Miravex Ltd., Dublin, Ireland). The results revealed a significant reduction in mean pore volume and area for the 1:10 dilution at the 2-week and 1-month follow-ups (*p* < 0.05), compared to the baseline. No significant differences were observed beyond the 2-month follow-up for either dilution, as shown in Table S2 and Figure S1.

#### 2.2.6. Subjective Evaluation of Treatment Results from Photographs by Two Dermatologists

Overall, the majority of investigators were satisfied with the treatment outcome in both dilutions (Figure S2). This is consistent with the treatment outcome from both Hemi-mMASI and brown spots with VISIA^®^ imaging (Canfield Scientific, Inc., Parsippany, NJ, USA). For dilution 1:5, the highest improvement was observed at 2 months, with 29% of patients reporting marked improvement. At 6 months, the results remained consistent, with 28% showing mild to moderate improvement and 24% experiencing marked improvement. For dilution 1:10, moderate improvement peaked at 2 months (32%), while marked improvement steadily increased, reaching 23% at 6 months. Overall, both dilutions demonstrated sustained improvement, with dilution 1:10 showing slightly greater effectiveness at the 6-month follow-up.

#### 2.2.7. Subjective Evaluation of Treatment Results by Volunteers

**Figure 6:**
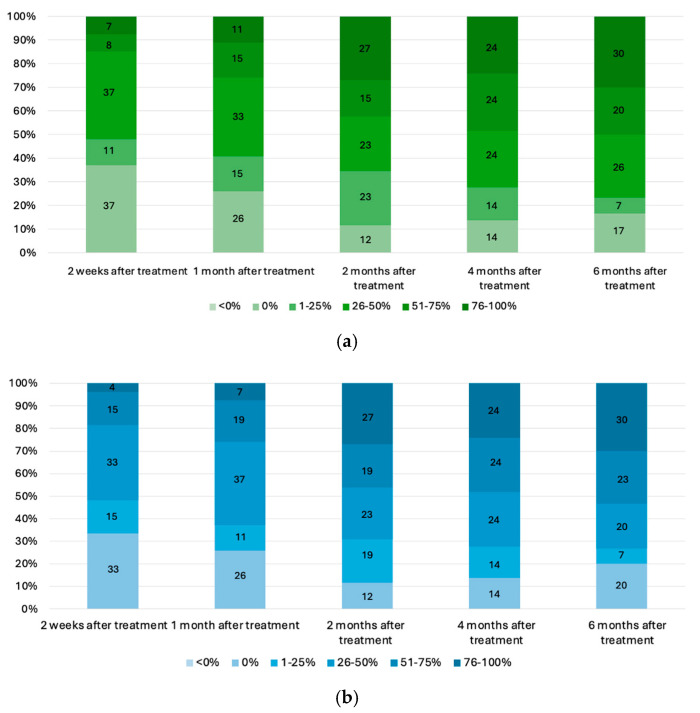
Patient Global Assessment (PGA) of melasma improvement in (**a**) 1:5 dilution and (**b**) 1:10 dilution.

Both dilutions show improvement over time, with a higher percentage of patients reporting 76–100% improvement at 6 months (30%) (Figure 6).

### 2.3. Adverse Events

Redness and dryness were reported by one patient two weeks after receiving the 1:10 dilution of Letibotulinum toxin A. Additionally, one patient experienced a transient asymmetrical grimace one month after treatment with the 1:5 dilution. Considering the overall efficacy and adverse event profile, the 1:10 dilution may be preferable, as it was not associated with any muscle-related complications and demonstrated a more favorable safety profile.

## 3. Discussion

This study demonstrates that both 1:5 and 1:10 dilutions of intradermal Letibotulinum toxin A significantly improve melasma severity. Improvements were observed across objective and subjective measures, including Hemi-mMASI scores, VISIA^®^ brown spot analysis, and both investigator- and patient-reported outcomes. Notably, the 1:10 dilution consistently yielded stronger statistical significance and more uniform improvements across endpoints, particularly in reducing pigmentation, brown spots, sebum levels, mean pore volume, and pore area. Importantly, no serious adverse events were reported in either group.

The primary aim of our study was to investigate the clinical effects of two different dilutions (1:5 and 1:10) utilizing a single commercially available product. LetiBoNT-A was selected as the study agent due to its increasing clinical utilization in Asia, including Thailand, as well as its established safety profile in aesthetic applications.

Hemi-mMASI scores improved significantly over the 6-month period with both dilutions (1:5, *p* = 0.043; 1:10, *p* = 0.002), while brown spot counts also significantly declined (1:5, *p* = 0.002; 1:10, *p* < 0.001). The 1:10 dilution showed statistically significant improvement as early as two weeks (*p* = 0.006), indicating a faster onset of action. Despite these trends, no statistically significant differences were found between dilutions at any time point, suggesting that both concentrations are clinically effective, though the 1:10 dilution may offer more rapid and sustained outcomes.

In terms of pore-related parameters, the 1:10 dilution demonstrated a significant decrease in mean pore volume and area at 2 and 4 weeks (*p* < 0.05). However, these improvements were not maintained beyond 2 months, suggesting the potential need for booster or maintenance treatments to prolong the effects. Sebum production also decreased significantly at 1 month with the 1:10 dilution (*p* = 0.037), reinforcing its efficacy in improving overall skin texture and oiliness.

It is important to recognize that the mechanisms of action of botulinum toxin type A (BoNT-A) on muscle, melanocytes, and sebaceous glands are mechanistically distinct. Therefore, the duration of BoNT-A’s effects in melasma cannot be presumed to parallel its duration in muscle relaxation or sebum suppression.

In skeletal muscle, BoNT-A acts by inhibiting acetylcholine (ACh) release at the neuromuscular junction, thereby preventing its binding to postsynaptic receptors and leading to functional denervation and muscle relaxation. This effect typically persists for 4–6 months, corresponding to the time required for axonal sprouting and synaptic reformation.

In contrast, cutaneous tissues express a non-neuronal cholinergic system (NNCS), whereby cells such as sebocytes, keratinocytes, and melanocytes synthesize and respond to ACh independent of neuronal input. Within sebaceous glands, ACh has been implicated in regulating sebocyte proliferation and sebum production. BoNT-A may attenuate sebum secretion by interfering with this cholinergic signaling; however, the in vivo response of human sebaceous glands to cholinergic blockade remains incompletely characterized. Notably, it is unclear whether chronic ACh exposure or receptor desensitization occurs with time. In our study, the reduction in sebum production following BoNT-A injection was significant but transient, persisting for approximately one month—considerably shorter than the duration typically observed in muscular effects.

Melanocytes, the primary pigment-producing cells, also express ACh receptors, suggesting that BoNT-A may modulate melanogenesis via cholinergic pathways. In vitro studies have demonstrated that BoNT-A reduces melanocyte dendricity and intracellular melanin content. In vivo treatment with BoNT-A has been associated with decreased pigmentation and reductions in DOPA-positive melanocytes, tyrosinase activity, melanin levels, basic fibroblast growth factor, interleukin-1 alpha, and prostaglandin E2. These data suggest a multifactorial inhibitory effect of BoNT-A on pigment production and inflammation [35].

Given these mechanistic distinctions, the duration and consistency of BoNT-A’s effects in melasma may differ from its muscular and sebaceous applications. Notably, the suppressive effects on melanogenesis and vascular proliferation, central to melasma pathophysiology, may be more durable due to broader immunomodulatory and anti-inflammatory actions. However, further studies are needed to elucidate the longevity of these effects and their underlying molecular mechanisms.

Subgroup analysis between melasma subtypes revealed that both telangiectatic and non-telangiectatic melasma improved in response to treatment. The telangiectatic group showed greater improvements with the 1:10 dilution, especially at 4 and 6 months, indicating potential long-term benefits in this subgroup. Telangiectatic melasma, which involves both pigmentary and vascular abnormalities, may particularly benefit from BoNT-A’s dual action on melanogenesis [14,16,18] and vascular modulation [21,36]. Studies using Dynamic Optical Coherence Tomography (D-OCT) have shown increased papillary and reticular dermal blood flow in melasma, especially within telangiectatic lesions [37]. The enhanced diffusion of the 1:10 dilution may allow for broader dermal penetration, potentially reaching vascular targets more effectively. These findings highlight the importance of considering melasma subtype and dilution characteristics when designing treatment protocols.

Although our study suggests that the 1:10 dilution offers not only greater efficacy but also an earlier onset of visible improvement, making it a promising option for patients seeking faster results, the question of treatment longevity remains important. Both dilutions demonstrated sustained improvement over the 6-month follow-up period, with the 1:10 dilution showing greater consistency across time points. For patients who achieve moderate improvement (51–75%) or those with telangiectatic melasma, additional treatment sessions may offer incremental benefits. Given the favorable safety profile observed, incorporating maintenance treatments at extended intervals (e.g., every 3–4 months) could be a practical strategy to maintain and enhance therapeutic outcomes.

Moreover, there was an improvement in adjacent untreated areas, such as the upper eyelids, as shown in Figure 2. BoNT-A may influence pigmentation beyond the injection site via indirect mechanisms. These include modulation of toll-like receptor 2 (TLR2)-mediated inflammation [38], cholinergic vasodilation [39,40], and neuropeptide signaling, particularly involving calcitonin gene-related peptide (CGRP) and substance P (SP). CGRP has been shown to upregulate NK-1R, which cooperates with SP to inhibit tyrosinase activity and melanin synthesis in melanocytes—even outside directly treated regions [41,42].

While previous studies have indicated that factors such as injection volume, concentration, and dosage play a significant role in toxin diffusion, whereas molecular weight and the presence of complexing proteins appear to have minimal impact, our findings may not be extrapolated to other BoNT-A products. Both preclinical and clinical data suggest that botulinum toxin formulations are not interchangeable, with some products demonstrating greater diffusion properties and higher rates of diffusion-related adverse events. This variability is attributed to the fact that each botulinum toxin product constitutes a unique pharmacological entity. Accordingly, a comprehensive understanding of the individual characteristics and diffusion profiles of different BoNT-A formulations is essential for accurately assessing their clinical effectiveness and for minimizing the risk of unintended diffusion and associated adverse effects [43,44,45].

Adverse events were minimal. One patient in the 1:10 group reported mild redness and dryness, which resolved spontaneously. One case of transient facial asymmetry occurred in the 1:5 group, which resolved without intervention. The absence of muscle-related side effects in the 1:10 group supports its favorable safety profile, particularly in aesthetic applications where symmetry is essential. Lastly, although our study demonstrated minimal side effects, it is important to note that injections were limited to the cheek area. In cases where melasma is presented more diffusely, caution should be exercised. It would be valuable to define clear safety margins, particularly by avoiding the upper and medial areas near the periorbital region, the nasolabial fold, and the corners of the mouth, to minimize the risk of unintended effects on facial muscles.

## 4. Conclusions

The 1:5 and 1:10 dilutions of Letibotulinum toxin A significantly improved melasma severity after a single treatment. Compared to the 1:5 dilution, the 1:10 dilution provided faster and more sustained improvements in pigmentation, skin texture, and sebum production, making it the better option for patients desiring quicker results. Although not statistically significant, the telangiectatic melasma subgroup showed greater proportional improvement with the 1:10 dilution at 6 months. Additionally, the 1:10 dilution may reduce the risk of asymmetry and offers a safer profile, supporting its potential for use in ongoing or combination therapies.

## 5. Materials and Methods

### 5.1. Study Design and Patient Selection

This was a randomized double-blind split-face clinical trial conducted at a university hospital in Bangkok, Thailand. A total of 30 Thai participants (29 women, 1 man; mean age 47 ± 6.6 years; range 32–62) with epidermal or mixed-type melasma and Fitzpatrick skin types III–V were enrolled. This study aimed to compare the efficacy and safety of two intradermal dilutions of Letibotulinum toxin A (LetiBoNT-A) for melasma treatment. The trial was registered in the Thai Clinical Trials Registry (TCTR20250208002; https://www.thaiclinicaltrials.org/show/TCTR20250208002, accessed on 1 July 2025), with the first submission date on 12 January 2025. The sample size was estimated based on a prior study [33], which showed a reduction in melanin index after 8 weeks of intradermal botulinum toxin A. With a two-sided alpha of 0.05 and 80% power, 26 participants were required. Accounting for a 15% dropout, the target sample size was 30. No changes were made after the trial commenced.

Recruitment of follow-up participants was conducted between January and June 2024, with follow-up completed by December 2024. One participant was lost to follow-up due to scheduling conflicts. Thus, data from 30 participants were included in the final analysis. For those unable to attend in-person visits, the follow-up data were collected via telephone interviews [46] (Figure S3).

Key exclusion criteria included pregnancy, breastfeeding, recent BoNT-A or aesthetic procedures (within 6–12 months), autoimmune disease, coagulation disorders, systemic or topical pigment-modulating therapies within 1–6 months, recent facial laser treatments, and use of photosensitizing medications. Participants engaging in regular unprotected sun exposure were also excluded. This study was conducted according to the guidelines of the Declaration of Helsinki and was approved by the Ethics Committee of the Siriraj Institutional Review Board (si 457/2023) on 9 June 2023. Informed consent was obtained from all participants.

### 5.2. Treatment Preparation, Area Selection

**Figure 7:**
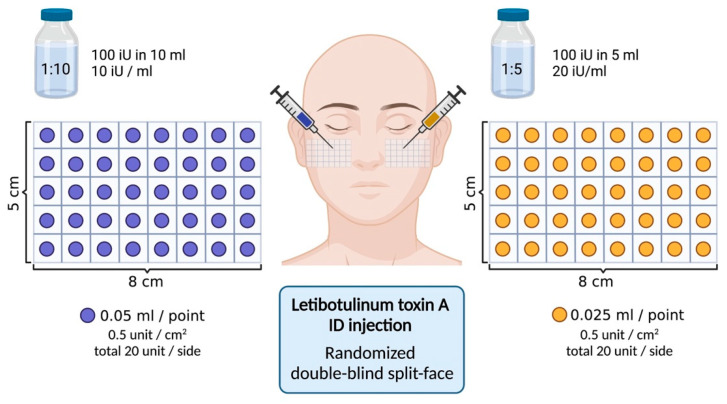
Location and amount of Letibotulinum toxin A injections.

LetiBoNT-A (Aestox^®^, Hugel Inc., Chuncheon, South Korea) was reconstituted into two concentrations: 1:5 dilution, 100 units in 5 mL normal saline (20 U/mL); and 1:10 dilution, 100 units in 10 mL normal saline (10 U/mL). Each side of the face was randomly assigned to receive one of the two dilutions using a computer-generated sequence (www.randomization.com, accessed on 1 July 2025). Injections were administered intradermally using a 30 G needle at a 20–30° angle over a standardized 40 cm^2^ cheek area (8 × 5 cm). A total of 20 units was injected per side at 0.5 units/cm^2^. The 1:5 dilution was injected at 0.025 mL/cm^2^, and the 1:10 dilution at 0.05 mL/cm^2^ (Figure 7). Topical anesthetic EMLA^®^ (AstraZeneca AB, Södertälje, Sweden) was applied 40–60 min before treatment.

To avoid asymmetries, it is essential to perform strictly intradermal injections while avoiding the zygomaticus minor and major muscles, both ideally located 1 cm from the orbital rim. The risorius muscle, important for lateral lip movement, should be injected approximately 2 mm lateral to the commissure to prevent uneven expression. Additionally, injections into the Levator Labii Superioris (LLS) and Levator Labii Superioris Alaeque Nasi (LLSAN) should be made 2 mm lateral to the alar-facial groove at the level of the nasal passage, ensuring proper muscle function and aesthetic outcomes [47,48,49,50].

The allocation sequence was concealed from the treating physician. Syringes were pre-filled and labeled by an independent assistant to ensure blinding. The injector, participants, and outcome assessors were blinded to treatment allocation.

### 5.3. Post-Treatment Care

Following treatment, the participants received ice compresses for 15–20 min. The participants were instructed to apply the provided broad-spectrum sunscreen daily and avoid any additional aesthetic procedures throughout the 6-month follow-up period. The use of other pigment-targeting therapies—including topical bleaching agents (e.g., alpha arbutin, AHA, vitamin C, azelaic acid, kojic acid, hydroquinone, and tretinoin), oral medications, injectable treatments, laser therapies, or energy-based devices—was strictly prohibited in the treated area for the duration of this study and until the final follow-up visit.

### 5.4. Objective Evaluation

For objective evaluations, standardized facial photographs were taken at baseline and all follow-up visits (2 weeks, 1, 2, 3, and 6 months) from five angles (90°, 45°, 0°, −45°, and −90°) under identical lighting and settings. Blinded dermatologists assessed clinical improvement using the Hemi-modified Melasma Area and Severity Index (Hemi-mMASI), calculated as follows:

*Hemi-mMASI* = 0.15 × *A(F)* × *P(F)* + 0.3 × *A(Malar)* × *P(Malar)* + 0.05 × *A(Mandibular)* × *P(Mandibular)*

where area (*A*) and pigmentation (*P*) are scored using established scales (0–6 and 0–4, respectively) [51].

Additional objective assessments included VISIA^®^ (Canfield Scientific, Inc., USA) for brown and red spot analysis, Antera 3D^®^ (Miravex, Dublin 2, Ireland) for mean pore area and volume over 5 cm^2^, and Sebumeter^®^ SM815 (Courage + Khazaka electronic GmbH, Köln Germany) to measure sebum levels after 20 min of acclimatization at 25 °C and 70% humidity.

### 5.5. Subjective Evaluation

Subjective clinical improvement was evaluated using the Investigator Global Aesthetic Improvement Scale (IGAS) and the Patient Global Assessment (PGA) grading scale, ranging from −1 (worsened) to 4 (excellent improvement: 76–100%). Two blinded dermatologists independently assessed standardized photographs at each visit (baseline, 2 weeks, 1, 2, 3, and 6 months). Disagreements were resolved by averaging scores. Participants also self-evaluated their improvement at each follow-up. Pain during injection was recorded using a 10-point Visual Analog Scale (VAS), and adverse events were assessed throughout this study, including erythema, bruising, irritation, dyschromia, and muscle paralysis.

### 5.6. Statistical Analysis

Data were analyzed using SPSS version 18.0 (IBM Corp., Armonk, NY, USA). Descriptive statistics were reported as mean values ± standard deviations (SD) for continuous variables and as frequencies and percentages for categorical variables. The Shapiro–Wilk test was used to assess normality.

For within-group comparisons over time, repeated-measures ANOVA was applied for normally distributed data, and the Friedman test was used for non-parametric data. For between-group comparisons, paired *t*-tests or Wilcoxon signed-rank tests were performed, depending on data distribution. A *p*-value < 0.05 was considered statistically significant.

## 6. Study Limitations

This study was limited by its relatively small sample size and its focus on Thai patients with Fitzpatrick skin types IV–V, potentially limiting its generalizability to other populations. Future studies should include more diverse cohorts and longer follow-ups. Incorporating advanced objective imaging tools and exploring combination or comparative therapies could further refine our understanding of BoNT-A’s role in melasma treatment.

## Figures and Tables

**Table 1:** Demographic data of subjects enrolled in this study.

Characteristics		Value (*n* = 30)
Age in years		
Mean ± SD * (min–max)		47 ± 6.60 (32–62)
Sex, *n* (%)		
Male		1 (3%)
Female		29 (96.67%)
Fitzpatrick Skin type, *n* (%)		
Type III		4 (13.33%)
Type IV		21 (70%)
Type V		5 (16.67%)
Melasma type, *n* (%)	Non-telangiectatic	16 (53.33%)
	Telangiectatic	14 (46.67%)
Baseline Hemi-mMASI score		
	1:5 dilution	4.44 ± 1.82
	1:10 dilution	4.66 ± 1.85
Baseline Hemi-mMASI score		
Non-telangiectatic	1:5 dilution	3.78 ± 1.45
	1:10 dilution	3.95 ± 0.90
Telangiectatic	1:5 dilution	5.20 ± 1.96
	1:10 dilution	5.48 ± 2.30
Mean pain score		
Mean ± SD * (min–max)	1:5 dilution	5.13 ± 2.29 (min = 0, max = 9)
	1:10 dilution	5.23 ± 2.21(min = 0, max = 9)

* SD, standard deviation.

**Table 2:** Mean Hemi-modified Melasma Area and Severity Index.

Follow-Up	Mean Hemi-mMASI (1:5)	Mean Hemi-mMASI (1:10)	*p*-Value Comparing Two Concentrations
Baseline	4.38 ± 1.66	4.47 ± 1.63	
2-week follow-up	4.16 ± 1.69 (*p* = 0.158)	4.06 ± 1.48 (*p* = 0.006) **	>0.99
1-month follow-up	3.87 ± 1.46 (*p* = 0.049) *	3.94 ± 1.22 (*p* = 0.082)	>0.99
2-month follow-up	3.95 ± 1.60 (*p* = 0.080)	3.84 ± 1.45 (*p* = 0.013) *	>0.99
4-month follow-up	3.97 ± 1.59 (*p* = 0.100)	3.70 ± 1.39 (*p* = 0.002) **	0.847
6-month follow-up	3.78 ± 1.49 (*p* = 0.043) *	3.53 ± 1.32 (*p* = 0.002) **	>0.99

*p*-value < 0.05 (*), *p*-value < 0.01 (**). Statistical comparison was made versus baseline.

**Table 3:** Assessment of VISIA^®^ complexion analysis (Canfield Scientific, Inc., Parsippany, NJ, USA) percentage data.

Follow-Up	Brown Spots (1:5)	Brown Spots (1:10)	Red Areas (1:5)	Red Areas (1:10)
Baseline	17.93 ± 5.79	17.95 ± 5.61	13.68 ± 11.77	14.69 ± 11.43
2-week follow-up	16.88 ± 5.23	17.02 ± 5.19	14.15 ± 11.31	15.10 ± 11.25
	(0.131)	(0.203)	(>0.99)	(>0.99)
1-month follow-up	16.30 ± 4.99	16.41 ± 5.27	12.61 ± 11.32	14.09 ± 11.16
	(0.017) *	(0.007) **	(>0.99)	(>0.99)
2-month follow-up	16.24 ± 4.28	16.38 ± 4.87	12.40 ± 10.66	13.05 ± 10.35
	(0.001) **	(0.001) **	(>0.99)	(0.834)
4-month follow-up	16.11 ± 4.61	15.94 ± 4.69	11.68 ± 10.65	12.78 ± 10.14
	(0.010) *	(0.001) ***	(0.208)	(0.348)
6-month follow-up	15.64 ± 4.48	15.5 ± 4.71	11.76 ± 10.75	12.32 ± 10.45
	(0.002) **	(<0.001) ****	(0.227)	(0.032) *

*p* < 0.05 (*), *p* < 0.01 (**), *p* < 0.001 (***), *p* < 0.0001 (****). Statistical comparisons were performed versus baseline.

## Data Availability

The original contributions presented in this study are included in this article and Supplementary Materials. Further inquiries can be directed to the corresponding author.

## Supplementary Materials

The following supporting information can be downloaded at https://www.mdpi.com/article/10.3390/toxins17070349/s1: Figure S1. Pore volume and area assessed with Antera^®^. Figure S2. Investigator Global Aesthetic Improvement Scale (IGAS) of melasma improvement in (a) 1:5 dilution and (b) 1:10 dilution. Figure S3. CONSORT 2025 flow diagram. Table S1. Assessment of Sebumeter^®^. Table S2. Assessment of mean pore volume and mean pore area with Antera^®^. Video S1. A treatment procedure.
