# Supplementary material for: Efficacy and Safety of Letibotulinum Toxin A for the Treatment of Melasma in Two Different Dilutions: A Randomized Double-Blind Split-Face Study
Source: Toxins (Basel). 2025 Jul 11;17(7):349. doi: 10.3390/toxins17070349 (PMC12299615; doi:10.3390/toxins17070349)
Supplement: Supplementary file 1 [file toxins-17-00349-s001.zip › toxins-3723136-supplementary (1)/toxins-3723136-Supplementary Materials.pdf]

Supplementary Material

Table S1. Assessment of Sebumeter®

Follow-Up	Sebum level (1:5)	Sebum level (1:10)
Baseline	26.33 ± 28.89	29.13± 32.87
2-week follow-up	26.68 ± 25.65<br>(>0.99)	27.39 ± 29.08<br>(>0.99)
1-month follow-up	25.41±41.09<br>(>0.99)	14.37 ± 18.21<br>(0.037)*
4-month follow-up	18.64 ±23.87<br>(>0.99)	21.29 ± 29.71<br>(0.497)
6-month follow-up	16.38 ± 17.52<br>(0.151)	16.90 ± 18.79<br>(0.097)

**P*-value compared to baseline with statistically significant difference.

Table S2. Assessment of mean pore volume and mean pore area by Antera®

Follow-Up	Mean pore volume (1:5)	Mean pore volume (1:10)	Mean pore area (1:5)	Mean pore area (1:10)
Baseline	0.0070±0.0054	0.0078±0.0049	0.3660±0.1679	0.3919±0.1618
2-week follow-up	0.0064±0.0051<br>(0.272)	0.0068±0.0043<br>(0.0498)*	0.3379±0.1638<br>(0.157)	0.3513±0.1375<br>(0.029)*
1-month follow-up	0.0064±0.0058<br>(0.647)	0.0065±0.0037<br>(0.0420)*	0.3331±0.1836<br>(0.336)	0.3406±0.1230<br>(0.027)*
2-month follow-up	0.0067±0.0045<br>(>0.99)	0.0071±0.0040<br>(0.8050)	0.3539±0.1451<br>(>0.99)	0.3632±0.1264<br>(0.454)
4-month follow-up	0.0068±0.0052<br>(>0.99)	0.0068±0.0044<br>(0.4420)	0.3570±0.1856<br>(>0.99)	0.3593±0.1519<br>(0.503)
6-month follow-up	0.0069±0.0043<br>(>0.99)	0.0071±0.0038<br>(>0.99)	0.3627±0.1452<br>(>0.99)	0.3726±0.1341<br>(>0.99)

**P*-value compared to baseline with statistically significant difference

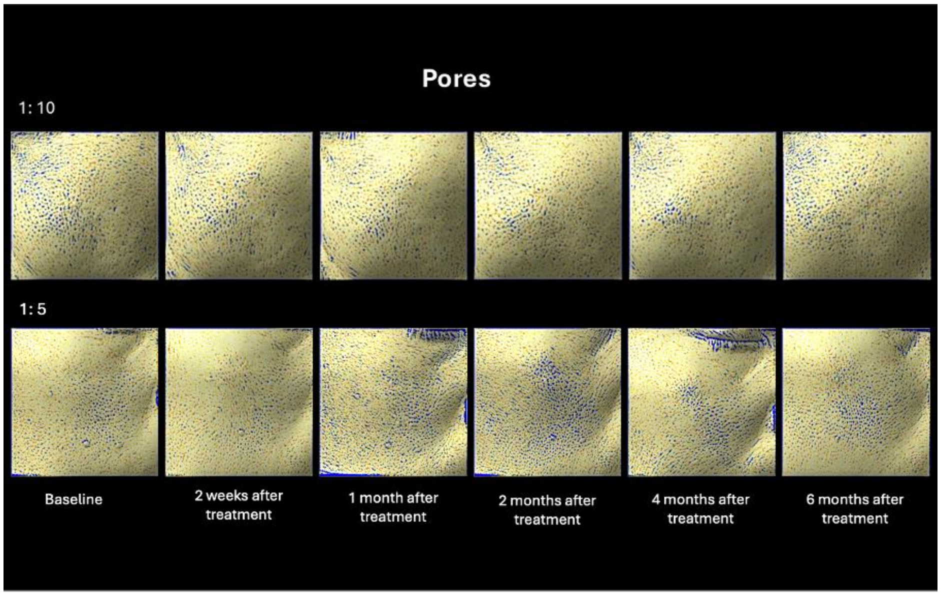

Figure S1. Pore volume and area assessed by Antera® : significant reduction in mean pore volume and area for the 1:10 dilution at the 2-week and 1-month follow-ups (p < 0.05)

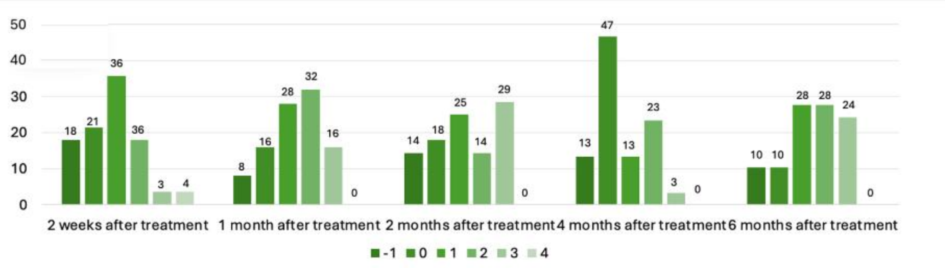

(a)

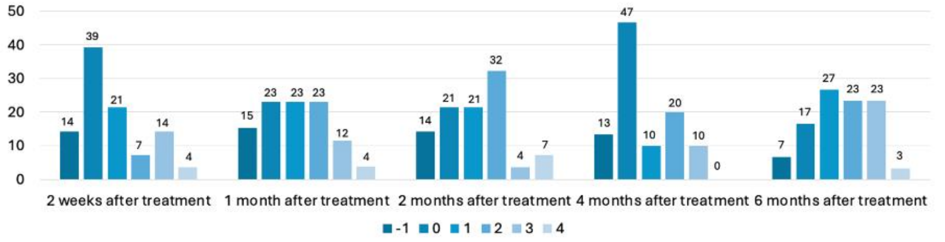

(b)

Figure S2. Investigator Global Aesthetic improvement scale (IGAS) of melasma improvement in (**a**) 1:5 dilution and (**b**) 1:10 dilution.

Diagram illustrating the progress of participants through the phases of this randomized, double-blind, split-face clinical trial. A total of 31 subjects were assessed for eligibility, enrolled, and randomized to receive intradermal botulinum toxin A injections at two different dilutions (1:5 and 1:10) on contralateral sides of the face. All participants received the assigned interventions. One participant was lost to follow-up due to scheduling conflicts and was excluded from the final analysis. Data from 30 participants (corresponding to 60 hemifacial units) were included in the final efficacy and safety assessments.

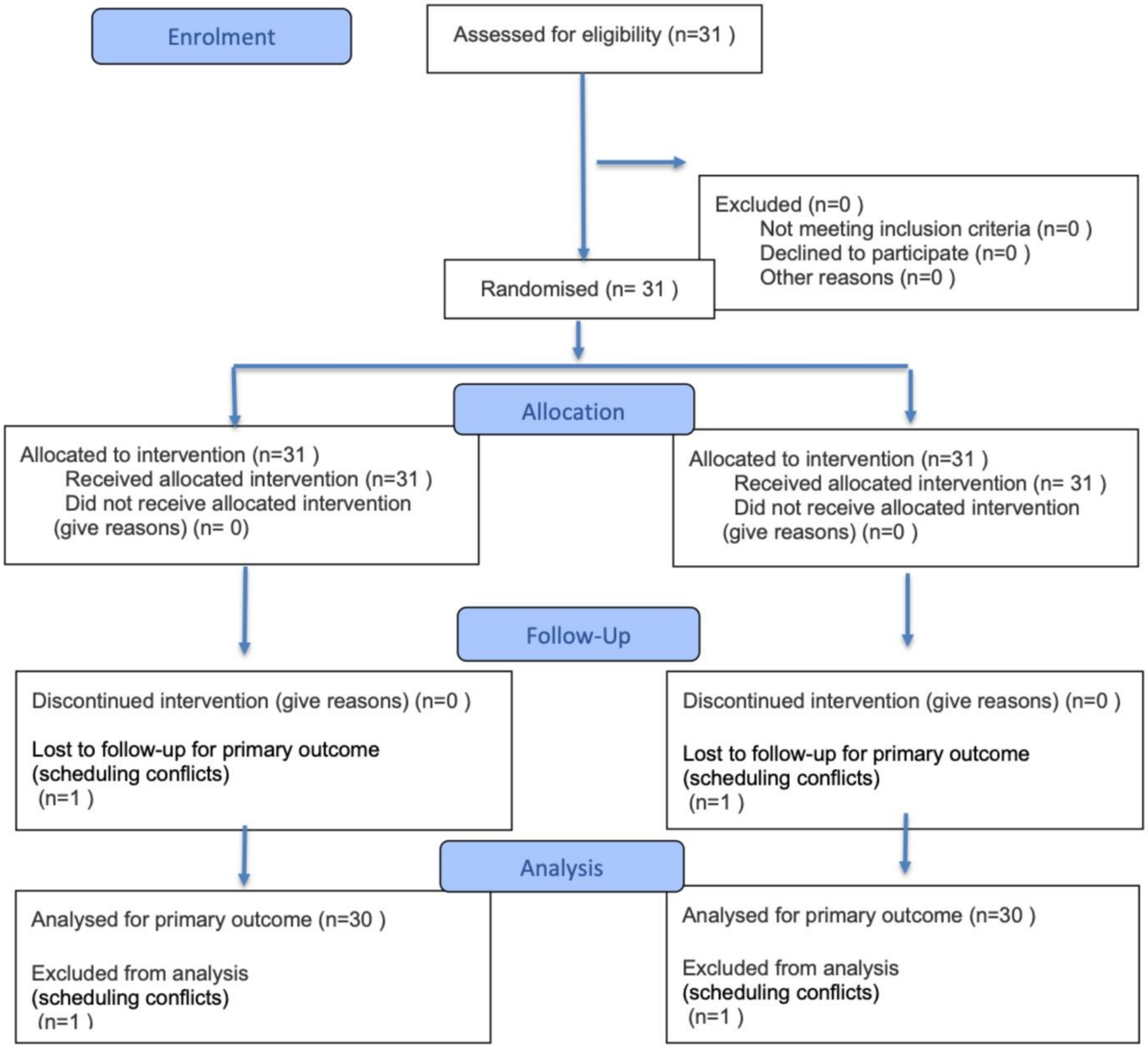

Figure S3. CONSORT 2025 Flow Diagram
